# Assessing transmission ratio distortion in extended families: a comparison of analysis methods

**DOI:** 10.1186/s12919-016-0030-0

**Published:** 2016-10-18

**Authors:** Sahir R. Bhatnagar, Celia M. T. Greenwood, Aurélie Labbe

**Affiliations:** 1 Department of Epidemiology, Biostatistics and Occupational Health, McGill University, 1020 Pine Avenue West, Montreal, QC H3A 1A2 Canada; 2 Lady Davis Institute for Medical Research, Jewish General Hospital, 3755 Côte Ste. Catherine, Montreal, QC H3T 1E2 Canada; 3 Departments of Oncology and Human Genetics, McGill University, Montreal, QC Canada; 4 Department of Psychiatry, McGill University, Montreal, QC Canada

## Abstract

A statistical departure from Mendel’s law of segregation is known as transmission ratio distortion. Although well documented in many other organisms, the extent of transmission ratio distortion and its influence in the human genome remains incomplete. Using Genetic Analysis Workshop 19 whole genome sequence data from 20 large Mexican American pedigrees, our goal was to identify potentially distorted regions in the genome using family-based association methods such as the transmission disequilibrium test, the pedigree disequilibrium test, and the family-based association test. Preliminary results showed an unusually high number of transmission ratio distortion signals identified by the transmission disequilibrium test, but this phenomenon could not be replicated by the pedigree disequilibrium test or family-based association test. Applying these tests to different subsets of the data, we found the transmission disequilibrium test to be very sensitive to imputed genotypes. Regression analysis of transmission ratio distortion test *p* values controlling for minor allele frequency and quality control checks showed that Hardy Weinberg *p* values are associated with this inflation. Although the transmission disequilibrium test appears confounded by imputation of single nucleotide polymorphisms, the pedigree disequilibrium test and family-based association test seem to offer more robust alternatives when searching for transmission ratio distortion loci in whole genome sequence data from extended families.

## Background

Transmission ratio distortion (TRD) refers to a significant departure from the expected Mendelian transmission of alleles from parents to offspring, which is why it can only be observed in family-based studies. Meiotic drive, gametic competition, embryo lethality, germline selection and imprinting errors are among the known biological processes that can lead to skewed transmission probabilities [1]. Although biased transmission from parents to children affected with a particular disease is a form of TRD by definition, in this study, we are concerned about the form of TRD that occurs in the general population that is unrelated to a specific disease. It is this form of TRD that can lead to spurious associations in genetic association studies of diseased populations. To test for this general form of TRD, ideally one needs families or trios unselected for phenotype or disease. Although we were aware that the Genetic Analysis Workshop 19 (GAW19) families were ascertained because of their type 2 diabetes, nevertheless, we thought that these data provided an opportunity to examine the frequency of sequence-identified variants displaying TRD. That is, even though these families are not the perfect choice to study TRD, they may still provide interesting insights [1]. The simplest test for detecting TRD is the transmission disequilibrium test (TDT) [2], which uses genotype information in trio families (2 parents, 1 affected offspring). To identify TRD in families unselected for disease, all offspring are considered as “affected,” which essentially means “having survived.” Therefore, the objective is to determine regions in the genome linked to the phenotype defined as “being alive in the last generation” [3].

Given the multigenerational structure of the GAW19 data combined with the availability of whole genome sequence data, our original objective was to evaluate the prevalence of TRD in sequencing-identified variation. After some preliminary analysis, however, we noticed that the imputation of genotypes for family members of sequenced individuals was creating strange patterns. For example, across odd-numbered chromosomes, the TDT identified more than 8000 single nucleotide polymorphisms (SNPs) with *p* values for TRD <10^−10^, compared to none less than this threshold when limiting the analysis to sequenced subjects only. To this end, we decided to explore possible reasons for this unexpected inflation by comparing other family-based methods of association including the pedigree disequilibrium test (PDT) [4] and the family-based association test (FBAT) [5, 6] across different subsets of the data.

## Methods

### Data

For our analysis, we worked with the sequenced/imputed genotype family data from GAW19 in the GENO files provided to the workshop [7]. We did not use the expression data. We also used the quality control variables stored in the variant call format (VCF) files to investigate the association between observed *p* values and variant call quality. Full details of the imputation procedure and quality control variables can be found in Almasy et al. [8].

One subject chosen at random from each of the 2 pairs of monozygotic twins was dropped from the analysis. The genotypes were provided free of Mendelian errors, and monomorphic loci were removed resulting in a total of 8,348,674 SNPs. We did not filter for significant departures from Hardy-Weinberg equilibrium (HWE) or small minor allele frequencies (MAFs) because we wanted to see if these features were associated with certain patterns in our results. We created a new dichotomous phenotype for hypertension where a subject was treated as “affected” if the subject had hypertension in any 1 of the 4 visits, resulting in 370 cases, 562 unaffected individuals, and 455 missing phenotypes. To compare the different family-based tests, we considered 3 subsets of the family data:

1. **All**: 1387 subjects, which consists of everyone minus the 2 randomly chosen monozygotic twins.
2. **Sequenced**: the 464 sequenced subjects.
3. **Nuclear**: 136 subjects consisting of 1 nuclear family per pedigree. This is a combination of 42 sequenced and 94 imputed subjects. We chose the largest possible family within each pedigree for which there was genotype information.

The rationale behind choosing the *all* and *sequenced* subsets was to see the effect imputation had on the test statistics. Furthermore, the *nuclear* subset was selected to facilitate comparison between the TDT, which breaks the families into trios and treats them as independent, with the PDT and FBAT, which account for the correlations between families in their test statistics.

### Family-based association analysis

We performed the TDT [2], PDT [4] and FBAT [5, 6] on each of 3 subsets of the GAW19 data just described. These methods look for transmission patterns to affected offspring. Therefore, the easiest way to test for TRD with existing software is to pretend that all children are “affected” [3]. The TDT was applied to the best called genotypes for the imputed data which were provided by the GAW19 organizers to all participants. TDT analysis was conducted in PLINK 1.9 [9] using the - tdt flag command. The PDT was implemented in the PDT software [4], with parameter option 0, which tells the program to use all information available. The FBAT analysis was performed with the FBAT software [5, 6] and specifying the -e flag which computes the test statistic using an empirical variance to account for the correlation between families [10]. Results were summarized in Manhattan plots created by the qqman package in R.

### Regression analysis

The objective of the regression analysis was to determine if any of the inflation observed in the FBAT *p* values was being explained by deviations from HWE, as measured by the *p* values from tests of HWE, while controlling also for the MAF at each SNP and the quality control measures extracted from the VCF files. Poor quality genotypes often display evidence of Hardy-Weinberg (HW) disequilibrium. For each test, we ran the following multiple linear regression model in the *sequenced* subset:

1 $$ -{ \log}_{10}(p)\sim -{ \log}_{10}\left({\mathrm{HWE}}_{p\mathrm{value}}\right) - { \log}_{10}(MAF)+ Quality\  Control\  Variables $$

and the following model for the *all* and *nuclear* subsets (since quality control data is only available for sequenced individuals):

2 $$ -{ \log}_{10}(p)\sim -{ \log}_{10}\left({\mathrm{HWE}}_{p\mathrm{value}}\right) - { \log}_{10}(MAF) $$

In the sequenced subset, we added quality control variables in case these parameters were also strongly associated with distribution of *p* values. We used the following quality measures from the VCF files (variable ID in parentheses): number of samples with fully called data (NS), strand bias Pearson’s correlation (STR), strand bias *z*-score (STZ), cycle bias *z*-score (CBZ), cycle bias Pearson’s correlation (CBR), base-quality inflation *z*-score (IOZ), ratio of base-quality inflation (IOR), alternate allele quality *z*-score (AOZ), alternate allele inflation score (AOI), and fraction of bases with map quality less than 10 (MQ10), less than 20 (MQ20), and less than 30 (MQ30). We first ran these models on all markers with *p* < 1, (*p* = 1 means all transmissions at the marker were noninformative). Then we restricted the regressions to markers with *p* < 10^−3^. Calculations were conducted in R.

## Results

### Family-based association tests

**Fig. 1:**
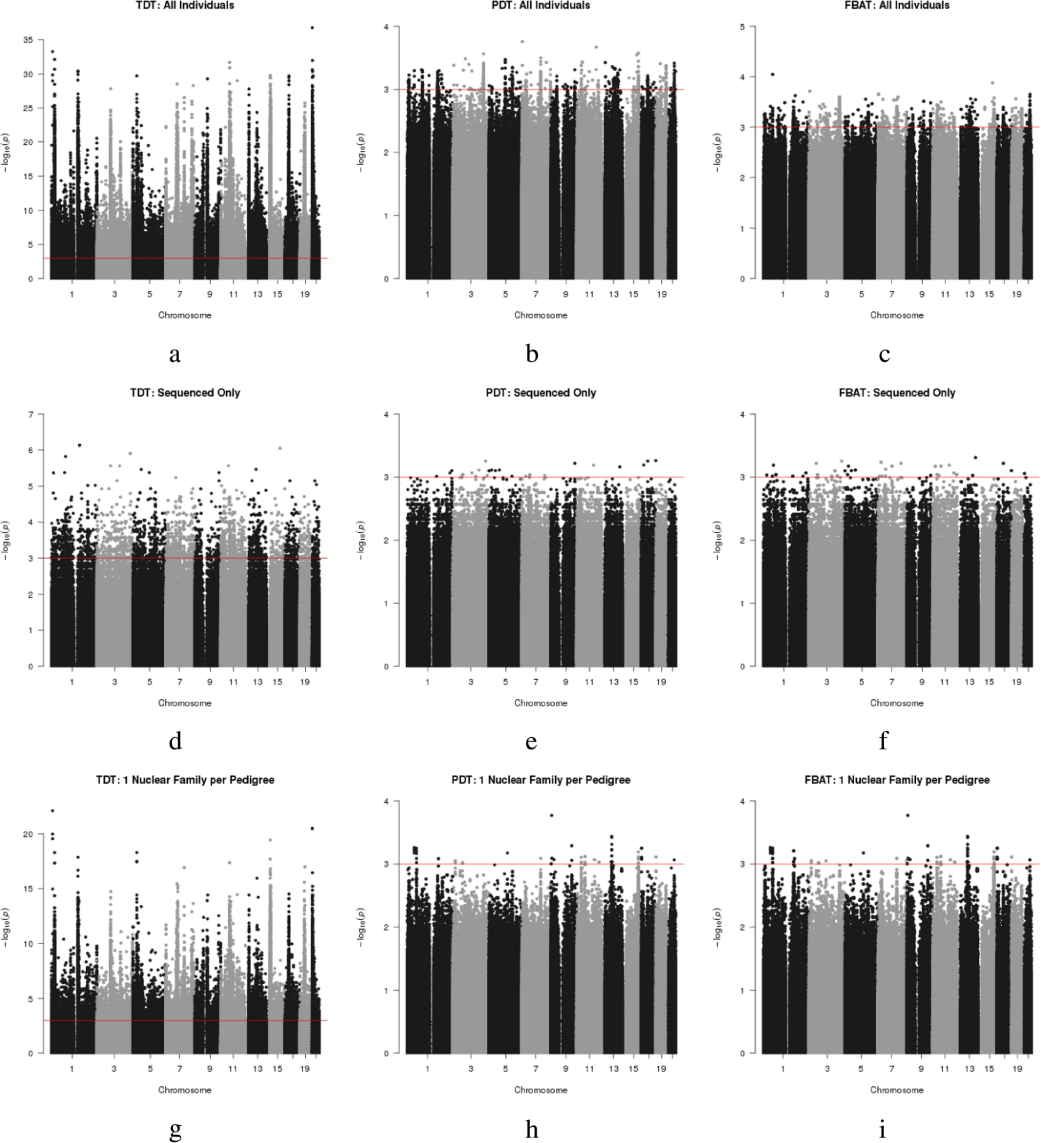
Manhattan plots for TDT (**a**, **d**, **g**), PDT (**b**, **e**, **h**) and FBAT (**c**, **f**, **i**) for all data (**a**, **b**, **c**), the sequenced subset (**d**, **e**, **f**), and the nuclear subset (**g**, **h**, **i**). Everyone has been coded as “affected” because we are evaluating evidence for general population TRD. A threshold line was set at 10^−3^ for comparability with the regression analysis. The effect of imputation on the test statistic can be seen by comparing across rows, while the impact of the different test statistics can be compared across columns. The *y*-axis varies across the plots

**Fig. 2:**
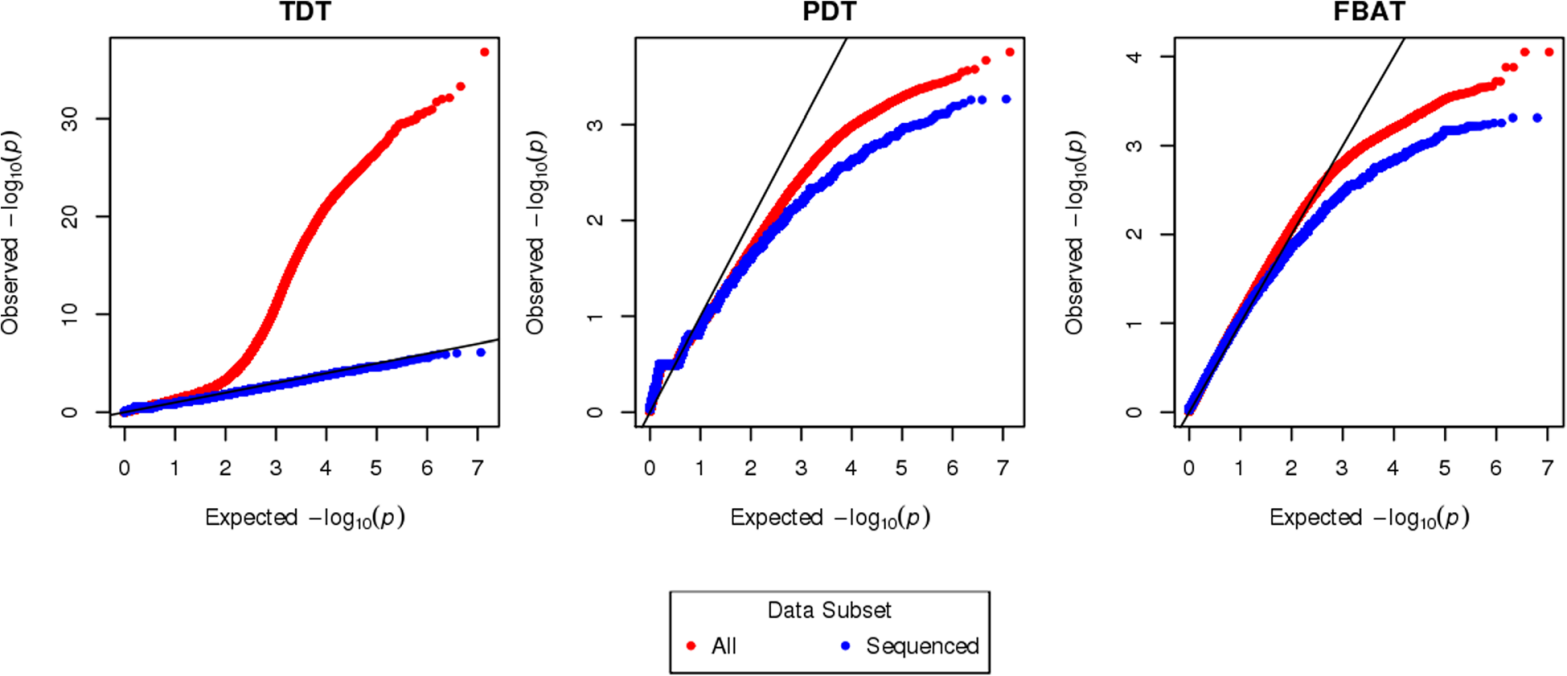
Q-Q plots of TDT, PDT, and FBAT *p* values for *all* (*red*) and *sequenced* (*blue*) subsets of the data. Both axes are plotted on the - log10 scale. The *y*-axis varies across the plots

Figure 1 summarizes the results from the family-based association analysis. We see that imputation of genotypes in nonsequenced individuals inflates the *p* value significance across all methods (e.g. Fig. 1a, d and g), although this is much more pronounced for the TDT. The Q-Q plots in Fig. 2 show that the distribution of TDT *p* values for the *all* subset is systematically different from what we would expect under the null, whereas the TDT *p* values for the *sequenced* subset of the data look as expected. The TDT also produces much smaller *p* values than PDT and FBAT, regardless of whether or not imputed individuals are included (e.g. Fig. 1d compared to Fig. 1e and f). The second and third columns of Fig. 1 depict the similarities between the PDT and FBAT in their performance and show that neither is very sensitive to imputation. The Q-Q plots for the PDT and FBAT (Fig. 2) show that these test statistics are overly conservative across *all* and *sequenced* subsets.

### Regression analysis

Table 1 shows the regression analysis results examining how much of the (−log10) *p* value variability for the TRD tests is explained by HW disequilibrium. When considering all SNPs, there is essentially no significant contribution of HW deviations to the ${R}^2$ for any test methods or for any subset of samples; however, we do see that HW disequilibrium contributes to ${R}^2$ differences when considering only SNPs with TRD *p* value< ${10}^{-3}$ for the *sequenced* and *nuclear* subsets. When looking at the marginal contribution of HWE test *p* values to the regression model, only the TDT rejected the null hypothesis of no contribution (in *F* tests) irrespective of the number of SNPs, method, and subset type considered. In the analysis of the sequenced subjects only, none of the quality control variables showed significant associations with the - log10 *p* values, probably because of good preprocessing of the sequence data.

**Table 1:** Contribution of HW disequilibrium to *p* value distribution, evaluated by regression analysis. Significant *F* tests (*p* < 0.05) demonstrating evidence of the contribution of HW disequilibrium to the *p* value distributions are shown in bold font

	All^b, c^			Sequenced^a, c^			Nuclear^b, c^		
Method	TDT	PDT	FBAT	TDT	PDT	FBAT	TDT	PDT	FBAT
Regression model of - log10 *p* values at all SNPs									
**R** ^**2**^_**full**_ **–R** ^**2**^_**reduced**_	4.4 × ${10}^{-4}$	3.6 × ${10}^{-7}$	1.5 × ${10}^{-6}$	0.0018	3.6 × ${10}^{-8}$	2 × ${10}^7$	2.9 × ${10}^{-6}$	8.26 × ${10}^{-9}$	3.5 × ${10}^{-7}$
# SNPs^e^	6.1 × ${10}^6$	5.6 × ${10}^6$	2.1 × ${10}^6$	3.2 × ${10}^6$	3 × ${10}^6$	8 × ${10}^5$	3.6 × ${10}^6$	2.1 × ${10}^6$	5 × ${10}^5$
Regression models of - log10 *p* values greater than 3 only									
**R** ^**2**^_**full**_ **–R** ^**2**^_**reduced**_	0.002	8.4 × ${10}^{-5}$	8.8 × ${10}^{-4}$	0.0015	${\mathrm{NA}}^f$	0.006	0.014	0.01	0.01
# SNPs^e^	8 × ${10}^4$	517	870	1777	11	19	3.3 × ${10}^4$	53	54
F-test *p*-values^d^									
All SNPs	**0.00**	0.16	0.07	**0.00**	0.74	0.69	**0.00**	0.89	0.68
*P*< ${10}^{-3}$	**0.00**	0.84	0.38	0.10	NA^f^	0.82	**0.00**	0.49	0.50

^a^Full model is given by Eq. (1)

^b^Full model is given by Eq. (2)

^c^Reduced model excludes $-{ \log}_{10}\left({\mathrm{HWE}}_{p\mathrm{value}}\right)$

^d^Test to see if there is a significant difference between the full model and the reduced model. Numbers presented correspond to *p*values of the *F* test where the null hypothesis is ${\beta}_{\mathrm{HWE}}=0$

^e^The number of informative SNPs

^f^Not enough data points to fit the model

## Discussion and conclusions

Although it is known that small levels of genotyping error can inflate TDT results [3, 6, 11], and that using a modal call as the best guess genotype leads to misclassification errors [12–15], our analysis shows that recently developed imputation algorithms giving no Mendelian errors [8] can have the same impact. These false-positive signals can occur for example from mistyping homozygote parents as heterozygotes (an error that is Mendelian consistent), or from missed calls among heterozygotes [11]. The majority of parent–child pairs were either not genotyped or discordant (one was genotyped and the other was not), which leaves a lot of room for imputation error. Our regression analysis showed that deviations from HWE explained only a small part of the variation in the TDT *p* values, indicating that these markers are not likely to be excluded on the basis of poor performance. Our results emphasize the caution that must be taken when using the TDT in the presence of imputed data. A more unexpected result was that the PDT and FBAT were not very sensitive to the presence of imputed data. One possible reason is that there are many informative nuclear family marker configurations that can contribute to the FBAT statistic [10] so it does not suffer from a severe loss of information when for example, parental genotypes are missing but sibship information is available and vice versa. The TDT would ignore this information. There are about 100 sib-pairs which would contribute accurately to the FBAT but would be completely ignored by the TDT, resulting in a smaller sample size and less power for the latter. Another possible explanation is in the way the variance of the test statistic is being calculated, that is, larger variances are resulting in smaller test statistics.

Because TRD was estimated by ignoring the true phenotype, we also performed these tests using our combined hypertension phenotype to see if different results would have been obtained. To do so, we repeated the analysis examining only transmissions to hypertensive individuals. The effect of the phenotype definition, that is, everyone affected versus hypertension, did not yield notable differences to the previous analyses for each method and subset type (results not shown).

**Fig. 3:**
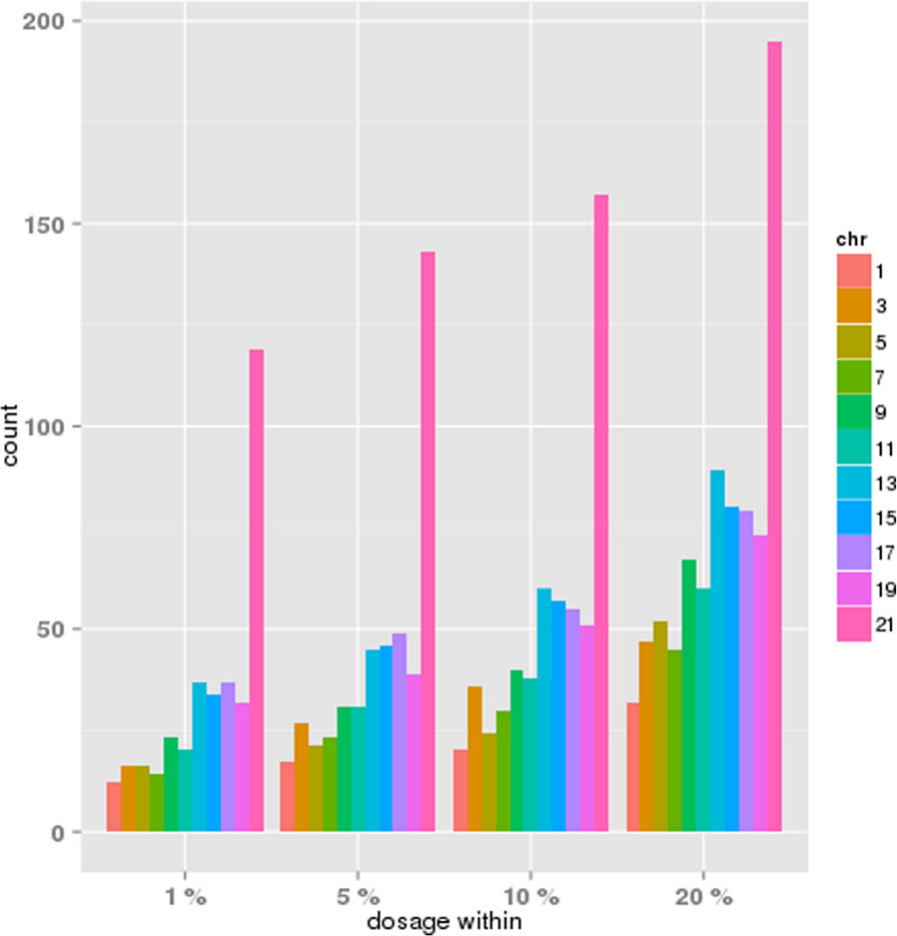
Number of subjects, by chromosome, where the imputed dosage genotypes were closer to an integer value at all markers than the indicated threshold

To further explore the effect of imputation, we examined the dosage files provided by GAW19 and extracted the “best” imputed individuals, that is, individuals whose genotype dosages were within 1, 5, 10 or 20 % of integer values 0, 1 or 2 (Fig. 3). We then repeated similar analyses, except we redefined the *all* subset to include only the individuals with these “best”-imputed genotypes, along with the sequenced subjects. This analysis showed much less inflation of TDT significance. Of note, we also observed significantly higher apparent dosage accuracy on chromosome 21. Specifically, on chromosome 21, dosage genotypes were with 1 % of an integer value for 119 individuals, whereas on other chromosomes, estimated dosages were within 1 % of integer values for only 12 to 37 individuals. Our findings therefore suggest that the TDT might be a useful measure of imputation quality for family data. For example, if the TDT reveals similar patterns to those in Fig. 1, this could be an indication that the data was poorly imputed.

Future work on this topic could focus on better understanding why imputation affects some methods more than others. It would be useful to run the same analysis on reimputed data that takes the pedigree structure into account during the prephasing step. A GAW19 contribution by Saad et al. showed that the prephasing algorithm has the greatest influence on imputation accuracy (for varying MAF), and that the best performing algorithm was one that accounted for family structure. We tried running TRD analysis on their best imputed data, though it did not yield any meaningful results as there were not enough informative families in that sample set.

Although it would be of interest to investigate whether any of our TRD signals are near any previously reported genes that are known to display TRD, it might be better to perform such analysis in data that are not imputed.
